# Comparison of methods for multivariate gene-based association tests for complex diseases using common variants

**DOI:** 10.1038/s41431-018-0327-8

**Published:** 2019-01-25

**Authors:** Jaeyoon Chung, Gyungah R. Jun, Josée Dupuis, Lindsay A. Farrer

**Affiliations:** 1 Bioinformatics Graduate Program, Boston University, Boston, MA USA; 2 Department of Medicine (Biomedical Genetics), Boston University School of Medicine, Boston, MA USA; 3 Neurogenetics and Integrated Genomics, Andover Innovative Medicines Institute, Eisai Inc, Andover, MA USA; 4 Department of Biostatistics, Boston University School of Public Health, Boston, MA USA; 5 Department of Neurology, Boston University School of Medicine, Boston, MA USA; 6 Department of Ophthalmology, Boston University School of Medicine, Boston, MA USA; 7 Department of Epidemiology, Boston University School of Public Health, Boston, MA USA

**Keywords:** Genetic association study, Risk factors

## Abstract

Complex diseases are usually associated with multiple correlated phenotypes, and the analysis of composite scores or disease status may not fully capture the complexity (or multidimensionality). Joint analysis of multiple disease-related phenotypes in genetic tests could potentially increase power to detect association of a disease with common SNPs (or genes). Gene-based tests are designed to identify genes containing multiple risk variants that individually are weakly associated with a univariate trait. We combined three multivariate association tests (O’Brien method, TATES, and MultiPhen) with two gene-based association tests (GATES and VEGAS) and compared performance (type I error and power) of six multivariate gene-based methods using simulated data. Data (*n* = 2000) for genetic sequence and correlated phenotypes were simulated by varying causal variant proportions and phenotype correlations for various scenarios. These simulations showed that two multivariate association tests (TATES and MultiPhen, but not O’Brien) paired with VEGAS have inflated type I error in all scenarios, while the three multivariate association tests paired with GATES have correct type I error. MultiPhen paired with GATES has higher power than competing methods if the correlations among phenotypes are low (*r* < 0.57). We applied these gene-based association methods to a GWAS dataset from the Alzheimer’s Disease Genetics Consortium containing three neuropathological traits related to Alzheimer disease (neuritic plaque, neurofibrillary tangles, and cerebral amyloid angiopathy) measured in 3500 autopsied brains. Gene-level significant evidence (*P* < 2.7 × 10^−6^) was identified in a region containing three contiguous genes (*TRAPPC12*, *TRAPPC12*-*AS1*, *ADI1*) using O’Brien and VEGAS. Gene-wide significant associations were not observed in univariate gene-based tests.

## Introduction

Genome-wide association study (GWAS) is a primary tool to identify association of genetic variants with phenotypes [1, 2]. GWAS has been successfully applied to a variety of complex diseases and identified genetic factors underlying complex diseases [3, 4]. However, there is still a considerable heritability of complex diseases that could not be explained by conventional GWAS [5, 6]. One plausible reason for unexplained heritability is due to the genetic architecture of complex diseases, which are affected by many common variants with low penetrance (i.e., small effect) [5]. Gene-based analysis, which considers the aggregate effect of multiple genic variants in a single test, is an alternative approach to overcome the genetic heterogeneity problem [7, 8]. Conventional GWAS may also be limited by phenotypic heterogeneity [5, 6, 9]. Most GWASs consider a univariate clinical outcome (e.g., disease diagnosis or a composite score of several disease-related traits). It is well understood that some variants may influence multiple traits associated with a single complex disease, but association of those variants may not be detected in a model with a broadly defined outcome [10]. Thus multiphenotype analysis, which simultaneously considers more than one phenotype pathologically or clinically related with the disease, may help identify additional disease-related genetic associations.

Several gene-based association methods [11, 12] and multivariate association methods [9, 13, 14] have been developed and successfully applied to GWASs of complex diseases. Recently, van der Sluis et al. developed a multivariate gene-based test (MGAS) [15] that combines the TATES [9] method for multivariate single-nucleotide polymorphism (SNP) association testing and the GATES method [8] for gene-based univariate association testing. In this study, we evaluated the statistical performance of combinations of multivariate association methods and gene-based association methods in various simulation models. The gene-based methods tested in this study include VEGAS [16] and GATES [8], which have been frequently used for analyzing common SNPs. The tested multivariate association methods were O’Brien [17], TATES [9], and MultiPhen [13]. These methods have been implemented in freely available standalone software or in an R library that accepts as input files produced by commonly used GWAS tools. The goal of this study is to provide guidance on how to optimally select multivariate gene-based association method for analyzing common variants given the correlation of phenotypes and genetic background (e.g., linkage disequilibrium [LD]).

## Methods

The information in detail about the multivariate and gene-based association methods are described in Table 1.

**Table 1:** Description of the methods for multivariate and gene-based association testing

Type	Method name	Input	Other requirements	Output
Multivariate association test	O’Brien [17, 18]	Genome-wide association summary Statistics (*β* and SE or *Z*)	Genome-wide summary statistics, other than subset of genome	SNP-level summary statistics of *Z* score and *P* value
	TATES [9]	SNP-level association summary statistics (*P* value only)	Individual-level phenotype data or correlation structure of phenotype data	SNP-level summary statistics of *P* value
	MultiPhen [13]	Individual-level genetic and phenotypic data	Missing data in any genetic or phenotypic data will reduce sample size for actual association tests	SNP-level summary statistics of *β*, SE, and *P* value
Gene-based association test	VEGAS [16]	SNP-level association summary statistics (*P* value only)	Individual-level genotypes for computing LD	*P* values for gene-level significance
	GATES [8]	SNP-level association summary statistics (*P* value only)	Individual-level genotypes for computing LD	*P* values for gene-level significance

### Approaches for multiphenotype association testing

The O’Brien method combines univariate test statistics (i.e. *Z* scores or *β*) of all SNPs from GWAS of multiple phenotypes to compute a test statistic for pleiotropic effect [17, 18] and is implemented in an R library, CUMP [19]. This method calculates a statistic assumed to follow a multivariate normal distribution with mean (combined *Z* scores of all SNPs) and covariance matrix of the multiple phenotypes. The covariance matrix among phenotypes can be approximated by the sample covariance matrix of the *Z* scores of all SNPs [17, 19].

The Trait-based Association Test that uses the Extended Simes procedure (TATES) was developed to detect effects across correlated traits measured in the same individuals using summary association statistics in the form of *P* value for each trait [9]. For each variant, the approach takes the minimum *P* value across a set of univariate tests carried out on each phenotype and then applies a weight to the *P* value to account for the number of phenotypes tested and their correlation. TATES requires univariate test statistics (i.e., *P* values) and a correlation matrix of the multiple phenotypes.

MultiPhen performs ordinal regression using an inverted model whereby the genotype or imputed SNP allele dosage is the outcome variable and the phenotypes are the predictors [13]. This program uses individual-level data (genotypes and phenotypes) for computing regression models, whereas the O’Brien method and TATES use summary statistics (*β* and SE or *P* values) and tests association between a SNP and a set of phenotypes by conducting likelihood ratio test for model fit, testing whether all regression coefficients in the model are jointly significantly different from zero. MultiPhen is an R package available from CRAN.

### Approaches for gene-based testing

The Gene-based Association Test using the Extended Simes procedure (GATES) computes a gene-based *P* value using SNP-based *P* values and correlations between SNPs (or pairwise LD information) in a gene [8]. The individual SNP *P* values are combined in a manner that appropriately controls for the effective number of independent SNPs in a gene. The effective number of independent SNPs is estimated from the eigenvalues of the square root of LD matrix.

The Versatile gene-based test for Genome-wide Association Studies (VEGAS) allows the SNP-based chi-square test statistics in a gene to be combined in a gene-based test statistic [16]. An empirical null distribution for this gene-based test statistic is obtained through a simulation of multivariate standard normal random vectors (*Z* statistics) with mean 0 and the correlations (or LD) between SNPs in a gene. The simulated gene-based test statistic is the sum of the squared *Z* statistics (with a chi-square distribution). The observed gene-based test statistic is the sum of chi-squares (converted from *P* values). The empirical gene-based *P* value is the proportion of simulated gene-based test statistics that surpass the observed gene-based test statistics. To compute the empirical gene-based *P* value, we performed 10^6^ simulations.

### Genotype simulation settings

Simulation studies under a range of scenarios were performed to assess and compare the performance (type I error and power) of the three multivariate association methods (O’Brien, TATES, and MultiPhen) each paired with one of the gene-based association methods (GATES and TATES). For all scenarios, we generated sequence genotypes of 22 autosomal chromosomes. HAPGEN2 software [20] was applied for generating the sequence genotypes for 2000 samples, and the European ancestry populations in the 1000 human genome reference panel (GRCh37; Mar 2012) [21] was used as reference to incorporate realistic genetic background. Only common SNPs with minor allele frequency (MAF) ≥1% were evaluated in the simulation tests for this study. For each simulation replicate, a 10-kb region containing at least 20 common SNPs was randomly selected. EIGENSTRAT [22] was used to generate principal components of the simulated genotypes to adjust for population structures in the simulation tests.

### Correlated multiphenotype simulation settings

Van der Sluis et al. suggested various genotype–phenotype models for genetic architecture of complex disease [9]. Among these models, we used the primary model, “single common factor model,” which implies that individual phenotypes related with a complex disease result from one shared latent factor influenced by genetic factors. Three correlated phenotypes of 2000 sample were simulated for each simulation test. The covariance matrix of three correlated phenotypes with the common factor were simulated according to this model:

$$\Sigma = \Lambda \ast \Lambda ^{\mathrm{T}} + \Theta ,$$

where *Σ* is the 3 × 3 covariance matrix among the three phenotypes, *Λ* is the 3 × 3 matrix of factor loadings, T is matrix transpose, and *Θ* is the 3 × 3 diagonal matrix of residual variances (see details elsewhere [9]). The factor loading is the proportion of variance of a complex disease explained by a factor, and the residual variance is the proportion of variance of phenotypes that is not explained by the factor. In general, when a factor loading value increases, the correlations among phenotypes simulated by the model above increase.

### Type I error simulation

We simulated three phenotypes that were multivariate normally distributed with mean 0 and covariance *Σ* for 2000 samples. Five million simulation tests were performed to evaluate whether the combined methods for multiphenotype gene-based test maintained the correct type I error rate. Type I error rate was evaluated through four scenarios by different factor loadings (*Λ* = 0.15, 0.35, 0.55, or 0.75, see Supplementary Table 1).

### Power simulation

To simulate multiphenotype data for the power evaluation, we randomly chose causal SNPs in the selected 10-kb region. We simulated three continuous phenotypes for 2000 samples using the formula

$$Y = \beta _1G_1 + \beta _2G_2 + \cdots + \beta _nG_n + \varepsilon ,$$

where *β*_*i*_ is the effect size of the causal SNP *i*, *G*_*i*_ is the genotype of the causal SNP *i*, and *ε* is error term that follows a multivariate normal distribution with means 0 and covariance matrix *Σ*. The effect size, *β*, was generated by

$$\beta _i = \sqrt {\frac{{h^2_q}}{{2 \times MAF_i \times (1 - MAF_i)}}} ,$$

where *h*^2^_*q*_, the proportion of variance explained by each causal SNP, was fixed at 1% for all scenarios and MAF_*i*_ is the MAF of the causal SNP *i*. We considered various scenarios in terms of different factor loadings (*Λ* = 0.15, 0.35, 0.55, and 0.75), i.e., different correlation between phenotypes and either 5% or 15% of causal SNPs as shown in Supplementary Table 1.

### Simulation test procedure

For each simulation replicate, multiphenotype association tests of SNPs were first conducted using the O’Brien, TATES, and MultiPhen approaches, and the association *P* values of SNPs from the three multivariate tests were combined into single gene-based *P* values using GATES and VEGAS. The O’Brien method requires genome-wide association statistics to compute a null distribution. To reduce the computation time, we generated a pruned set of uncorrelated SNPs on all chromosomes. The pruned SNP set was used for computing genome-wide association statistics, which were applied to compute the covariance matrix for the O’Brien approach. The full set of unpruned SNPs were used for the rest of simulation replicates. We used linear regression to compute univariate associations (*β*s and SEs or *P* values) between SNPs and each phenotype after adjusting the first three PCs, and the univariate SNP associations results were then used as input for analyses using the O’Brien and TATES multivariate association methods. MultiPhen computes multivariate associations with individual-level simulated data (SNPs and three phenotypes) after adjusting the three PCs. Because we simulated genotypes in the European ancestry population from the 1000 Genomes reference panel [21], the European LD structure from this panel (GRCh37; Mar 2012) was used for GATES and VEGAS to correct for the correlation between SNPs.

### Scenario setting

We investigated statistical performance, both type I error and empirical power, of each pair of multivariate gene-based association methods in various scenarios for four different factor loadings (*Λ* = 0.15, 0.35, 0.55, and 0.75) and proportions of independent SNPs in a gene. The effective number of independent SNPs in a gene was estimated in the manner applied in GATES [8], and proportions of independent SNPs out of the total number of SNPs in a gene were classified into three groups (low: <40%, moderate: 40–60%, and high: >60%).

We further considered additional scenarios for assessing the empirical power by varying (1) the percentage of causal variants (5% and 15%) in a gene, (2) phenotype direction (i.e., correlations of phenotypes are all in same direction or not), and (3) the number of phenotypes affected by the causal variants. We randomly selected 5% or 15% of causal variants among the total number of SNPs in a gene. We compared the empirical power of the paired multivariate gene-based association methods when all three phenotypes were positively correlated, with the sign of the pairwise correlation = (+++) or one phenotype was inversely correlated with the others (+−−). Also, we investigated the empirical power for the scenarios where the causal variants in a gene affect one or two phenotypes only, rather all three phenotypes. The simulation studies for the scenarios with varying phenotype correlation direction and the number phenotypes affected by causal variants were conducted with the factor loading fixed to 0.55. The complete range of simulation scenarios is summarized in Supplementary Table 1.

### Application to neuropathological traits related to Alzheimer disease (AD)

Genetic and phenotypic data for 3135 AD cases and 463 clinically and pathologically confirmed controls of European ancestry from 12 datasets (participant characteristics shown in Supplementary Table 2) who have genotypes and AD-related neuropathological phenotypes including neuritic plaque (NP), neurofibrillary tangles (NFT), and cerebral amyloid angiopathy (CAA) were obtained from the Alzheimer’s Disease Genetics Consortium. A total of 3598 subjects have both NP and NFT phenotype data, but only 2403 subjects have CAA data. GWAS of each of these three phenotypes has been conducted previously [23], and GWAS summary statistics of the three traits were obtained from NIAGADS (https://www.niagads.org) [24].

Neuropathological traits are correlated, but the correlations among traits vary, which is not covered by our simulations scenarios. Although our simulation study may provide sufficient information to infer statistical performance, especially type I error, of the proposed methods, we performed one more simulation study to assess the type I error for phenotypes with correlation structure similar to the neuropathological traits. An R multivariate normal generating (“mvrnorm”) function of the MASS library was applied to generate sets of phenotypes to mimic the neuropathological traits. The rest of the simulation setting were identical to the main simulation study.

Summary statistics of the three neuropathological phenotypes were used to evaluate the O’Brien and TATES multivariate association methods. The MultiPhen program was applied to each dataset and the SNP association results were combined across datasets using METAL with the weighted *Z*-score method based on sample sizes [25]. It should be noted that the sample size used for MultiPhen (# actual sample size for computation = 2403) is much smaller than the other multivariate association methods (O’Brien and TATES; # = 3135) because MultiPhen requires individual-level genotype and phenotype data (NP, NFT, and CAA). For the gene-based tests (VEGAS and GATES), we used SNPs within 10 kb of both ends of transcripts after removing low-frequency SNPs (MAF ≤ 1%) and SNPs with low imputation quality (*R*^2^ < 0.4). To evaluate the performance of the multivariate association methods in a consistent condition (i.e., the same subjects), we also performed a sensitivity analysis using a subset of individuals who had all genotype and no missing phenotype data (*n* = 2403).

The genome-wide significance level for gene-based tests was set as 2.7 × 10^−6^, which was calculated as the nominal significant level divided by the total number of genes tested (*n* = 18,500). We defined a gene having a pleiotropic effect on the three phenotypes when the multivariate gene-based *P* value was at least one order of magnitude lower than the univariate gene-based *P* value of each phenotype.

## Results

On average, 60 SNPs were observed in each gene region (10-kbp length). On average, correlation estimates (Pearson correlation, *r*) between phenotypes that were simulated by factor loadings (*Λ*) in scenarios were 0.01, 0.21, 0.57, and 0.86 for *Λ* = 0.15, 0.35, 0.55, and 0.75, respectively. Of note, simulated phenotypes were not significantly correlated (*P* > 0.05) when the factor loading was equal to 0.15.

### Type I error simulations

The empirical type I errors of multivariate association methods with the gene-based tests are shown in Table 2 (VEGAS) and Table 3 (GATES) at different *α* levels based on the proportion of independent SNPs in a gene (low, moderate, and high) and factor loading (*Λ* = 0.15, 0.35, 0.55, and 0.75). Applying VEGAS to the multivariate output by O’Brien (VEGAS-O’Brien) yielded slightly inflated type I errors at all *α* levels in the three scenarios by factor loadings (*Λ* = 0.15, 0.35, and 0.55) regardless of proportion of independent SNPs but not in the scenario of higher factor loading (*Λ* = 0.75). Applying VEGAS to rest of the multivariate association methods has inflated type I errors at *α* = 0.0001 for TATES and at all *α* levels for MultiPhen for all scenarios irrespective of factor loadings or independent SNP proportions. When GATES was applied, type I errors for all three multivariate association methods were deflated at all *α* levels in all scenarios.

**Table 2:** Type I error rate of multivariate association methods with a gene-based association method, VEGAS

Factor loading (*Λ*)	Proportion of independent SNPs (%)	VEGAS								
		*α* = 0.01			*α* = 0.001			*α* = 0.0001		
		O’Brien	TATES	MultiPhen	O’Brien	TATES	MultiPhen	O’Brien	TATES	MultiPhen
0.15	0–40	0.00	0.00	0.02	0	0.0005	0.001	0	0.0005	0.0002
	40–60	0.00	0.00	0.02	4.1 × 10^−5^	0.0004	0.003	1.4 × 10^−5^	0.0003	0.0005
	60–100	0.00	0.00	0.02	4.8 × 10^−5^	0.0002	0.003	0	0.0002	0.0007
0.35	0–40	0.00	0.00	0.02	0.0002	0.0006	0.002	0	0.0006	0.0002
	40–60	0.01	0.00	0.02	0.0002	0.0004	0.003	3.3 × 10^−5^	0.0004	0.0004
	60–100	0.01	0.00	0.02	0.0003	0.0003	0.003	5.7 × 10^−5^	0.0003	0.0006
0.55	0–40	0.01	0.00	0.02	0.002	0.0010	0.003	0.0001	0.0008	0.0002
	40–60	0.01	0.00	0.02	0.002	0.0005	0.003	0.0002	0.0004	0.0003
	60–100	0.02	0.00	0.02	0.001	0.0004	0.003	0.0002	0.0004	0.0006
0.75	0–40	0.06	0.00	0.02	0.01	0.0008	0.002	0.0015	0.0005	0.0006
	40–60	0.12	0.00	0.02	0.02	0.0007	0.003	0.0032	0.0006	0.0003
	60–100	0.19	0.00	0.02	0.03	0.0003	0.005	0.0071	0.0003	0.0009

**Table 3:** Type I error rate of multivariate association methods with a gene-based association method, GATES

Factor Loading (*Λ*)	Proportion of independent SNPs (%)	GATES								
		*α* = 0.01			*α* = 0.001			*α* = 0.0001		
		O’Brien	TATES	MultiPhen	O’Brien	TATES	MultiPhen	O’Brien	TATES	MultiPhen
0.15	0–40	0.000	0.006	0.010	0.0000	0.0004	0.0000	0.0000	0.0001	0.0001
	40–60	0.000	0.003	0.000	0.0000	0.0004	0.0000	0.0000	0.0000	0.0000
	60–100	0.000	0.002	0.000	0.0000	0.0002	0.0000	0.0000	0.0000	0.0000
0.35	0–40	0.000	0.006	0.010	0.0000	0.0008	0.0000	0.0000	0.0001	0.0001
	40–60	0.000	0.003	0.000	0.0000	0.0003	0.0010	0.0000	0.0000	0.0001
	60–100	0.000	0.002	0.000	0.0000	0.0001	0.0000	0.0000	0.0000	0.0000
0.55	0–40	0.002	0.006	0.010	0.0000	0.0009	0.0000	0.0000	0.0001	0.0001
	40–60	0.001	0.004	0.010	0.0000	0.0004	0.0000	0.0000	0.0000	0.0000
	60–100	0.001	0.002	0.000	0.0000	0.0002	0.0000	0.0000	0.0000	0.0000
0.75	0–40	0.010	0.008	0.010	0.0000	0.0005	0.0010	0.0000	0.0000	0.0000
	40–60	0.010	0.005	0.000	0.0000	0.0005	0.0000	0.0000	0.0001	0.0000
	60–100	0.000	0.002	0.000	0.0000	0.0001	0.0000	0.0000	0.0000	0.0000

### Power simulations

**Fig. 1:**
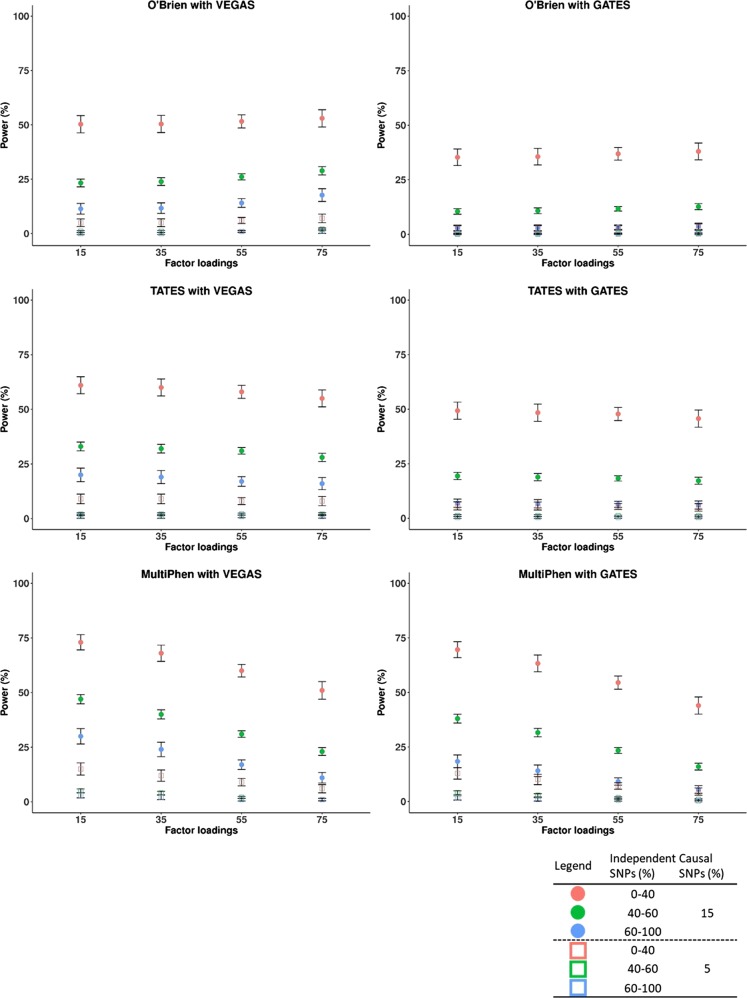
Power comparisons of multivariate association methods (O’Brien, TATES, and MultiPhen) with gene-based association methods (VEGAS and GATES) in various scenarios by varying the proportion of independent SNPs in a gene. **a** Causal variant percentage = 15% and **b** causal variant percentage = 5%. Empirical power calculated at *α* level of 0.0001

Power simulation results of multivariate association methods with gene-based association methods are shown in Fig. 1 and Supplementary Tables 3 and 4. As the proportion of independent SNPs in a gene increased, the power of all multivariate association methods decreased regardless of gene-based association method. Most of the multivariate association methods, except for MultiPhen, using VEGAS or GATES showed low power (<10%) in scenarios when the proportion of causal SNPs in a gene is equal to 5%. In the scenarios for the proportion of causal SNPs fixed at 15%, the power of O’Brien and TATES, regardless of the gene-based association methods, remained almost constant in the scenarios when factor loading (*Λ*) increases from 0.15 to 0.35, 0.55, and 0.75. In contrast, the power of MultiPhen combined with VEGAS or GATES was highest in the scenario including the lowest factor loading (*Λ* = 0.15), and it was reduced by about 20% when the factor loading increased from 0.15 to 0.75. All pairs of multivariate and gene-based association methods had similar power in the scenarios of the highest factor loading (*Λ* = 0.75). Applying VEGAS to the three multivariate association methods showed slightly greater power than GATES in all scenarios. However, this might be related to the inflated type I errors observed in most of the multivariate association methods using VEGAS.

In the scenario with phenotypes positively and negatively correlated (+−−), O’Brien combined with both GATES and VEGAS yielded low or zero power (Table 4a), while the empirical power of TATES and MultiPhen, regardless of the gene-based association methods, remained almost constant compared to the scenario with positively correlated phenotypes (+++; Table 4b). We also observed that, as the number of phenotypes not affected by causal variants increased, the power of all multivariate association methods decreased regardless of gene-based association methods (Table 5). This can be seen most clearly with the results of the O’Brien method paired with both gene-based association methods, where power is very low when the effect of causal variants is on only one phenotype among the three phenotypes.

**Table 4:** Power of multivariate association methods (O’Brien, TATES, and MultiPhen) with gene-based association methods (VEGAS and GATES) for phenotypes in the same or different directions

Proportion of independent SNPs (%)	VEGAS									GATES								
	*α* = 0.01			*α* = 0.001			*α* = 0.0001			*α* = 0.01			*α* = 0.001			α = 0.0001		
	O’Brien	TATES	MultiPhen	O’Brien	TATES	MultiPhen	O’Brien	TATES	MultiPhen	O’Brien	TATES	MultiPhen	O’Brien	TATES	MultiPhen	O’Brien	TATES	MultiPhen
(a) All three phenotypes are correlated with each other in the same direction (+++)																		
0–40	0.81	0.82	0.80	0.65	0.69	0.70	0.52	0.58	0.60	0.63	0.74	0.76	0.47	0.59	0.64	0.37	0.48	0.55
40–60	0.62	0.60	0.58	0.40	0.43	0.42	0.26	0.31	0.31	0.32	0.45	0.47	0.19	0.28	0.32	0.12	0.18	0.23
60–100	0.49	0.45	0.42	0.26	0.28	0.26	0.14	0.17	0.17	0.14	0.25	0.26	0.06	0.12	0.15	0.03	0.06	0.09
(b) One of the phenotypes is correlated with others in an opposite direction (+−−)																		
0–40	0.00	0.81	0.80	0.00	0.69	0.69	0.00	0.58	0.60	0.00	0.74	0.75	0.00	0.59	0.63	0.00	0.47	0.54
40–60	0.00	0.60	0.58	0.00	0.43	0.42	0.00	0.30	0.31	0.00	0.45	0.46	0.00	0.28	0.32	0.00	0.18	0.23
60–100	0.00	0.43	0.41	0.00	0.27	0.25	0.00	0.17	0.16	0.00	0.24	0.25	0.00	0.12	0.14	0.00	0.06	0.09

The factor loading and percentage of causal variants among the variants in a gene were fixed at 0.55 and 15%, respectively

**Table 5:** Power of multivariate association methods (O’Brien, TATES, and MultiPhen) with gene-based association methods ((a) VEGAS and (b) GATES)

# of phenotypes affected by causal variants	Proportion of independent SNPs (%)	*α* = 0.01			*α* = 0.001			*α* = 0.0001		
		O’Brien	TATES	MultiPhen	O’Brien	TATES	MultiPhen	O’Brien	TATES	MultiPhen
(a) VEGAS										
1	0–40	0.20	0.65	0.68	0.08	0.52	0.54	0.03	0.42	0.44
	40–60	0.08	0.38	0.41	0.02	0.24	0.41	0.01	0.24	0.18
	60–100	0.55	0.23	0.26	0.01	0.12	0.26	0.00	0.12	0.08
2	0–40	0.57	0.76	0.79	0.37	0.63	0.68	0.25	0.52	0.58
	40–60	0.34	0.52	0.56	0.15	0.36	0.41	0.08	0.25	0.30
	60–100	0.23	0.35	0.39	0.08	0.21	0.24	0.03	0.13	0.16
3	0–40	0.81	0.82	0.80	0.65	0.69	0.70	0.52	0.58	0.60
	40–60	0.62	0.60	0.58	0.40	0.43	0.42	0.26	0.31	0.31
	60–100	0.49	0.45	0.42	0.26	0.28	0.26	0.14	0.17	0.17
(b) GATES										
1	0–40	0.05	0.58	0.60	0.02	0.45	0.46	0.01	0.36	0.37
	40–60	0.01	0.28	0.30	0.00	0.17	0.18	0.00	0.11	0.12
	60–100	0.00	0.13	0.13	0.00	0.06	0.07	0.00	0.03	0.04
2	0–40	0.32	0.68	0.74	0.20	0.54	0.62	0.13	0.44	0.53
	40–60	0.10	0.38	0.45	0.04	0.24	0.31	0.02	0.15	0.22
	60–100	0.03	0.19	0.24	0.01	0.93	0.14	0.00	0.05	0.08
3	0–40	0.63	0.74	0.76	0.47	0.59	0.64	0.37	0.48	0.55
	40–60	0.32	0.45	0.47	0.19	0.28	0.32	0.12	0.18	0.23
	60–100	0.14	0.25	0.26	0.06	0.12	0.15	0.03	0.06	0.09

The factor loading and percentage of causal variants among the variants in a gene were fixed at 0.55% and 15%, respectively

Among the pairs of multivariate gene-based association methods that correctly controlled type I error, MultiPhen and GATES outperformed other combinations in most scenarios, suggesting that the pairing GATES with MultiPhen is an omnibus method for multivariate gene-based association testing.

### Application to AD-related neuropathological traits

The correlation estimates (*r*) between the three neuropathological phenotypes were 0.68 between NP and NFT, 0.56 between NP and CAA, and 0.40 between NFT and CAA. For the investigation of pleiotropic effects on the three neuropathological phenotypes, we planned to apply multivariate gene-based association methods that properly controlled the type I error in the simulation tests. From the extra simulation study for the phenotypes with correlation most similar to the neuropathological phenotypes, we found that the pairing of GATES with all three multivariate association methods maintained correct type I error. The method combined by O’Brien and VEGAS exhibited slightly inflated type I errors (0.0001–0.0003 at *α* = 0.0001), while the pairing of VEGAS with the other multivariate association methods (TATES and MultiPhen) showed substantial inflated type I error (0.0002–0.0008 at *α* = 0.0001; see Supplementary Table 5). Therefore, the pairing of GATES with all three multivariate association methods (O’Brien, TATES, and MultiPhen) plus the combination of VEGAS and O’Brien were used for analysis of the neuropathological phenotypes.

Association findings from multivariate gene-based methods are shown in Table 6 for previously reported AD genes [26–29] and Table 7 for new genes identified in this study with at least suggestive association (*P* < 10^−4^). Only 8 of the 27 previously known AD genes attained at least a nominally significant level of association with at least 1 of the phenotypes. Five of the previously reported AD genes—*BIN1*, *PICALM*, *TSPOAP1*, *CASS4*, and *APOE*—were at least nominally associated in the multivariate gene-based analyses at a significance level of at least one order of magnitude smaller than the results from the univariate analyses of three neuropathological phenotypes. *APOE* was detected as a gene with pleiotropy effect on the three phenotypes in the pairs of methods: GATES with O’Brien (*P* = 1.6 × 10^−68^) and TATES (*P* = 2.1 × 10^−44^). O’Brien with VEGAS also found the significant association for *APOE* (*P* < 1.0 × 10^−6^) in the multivariate gene-based analysis. However, O’Brien with VEGAS could not differentiate whether or not the multivariate association in *APOE* is more significant than its associations from each of univariate analyses because VEGAS generated a gene-based *P* value from a permutation approach that was not precise enough to detect a change of one order of magnitude. Nominally significant multivariate gene-based associations (*P* < 10^−3^) in *BIN1*, *PICALM*, and *CASS4* were observed from O’Brien with VEGAS, and nominal association in *TSPOAP1* was detected from MultiPhen with GATES (Table 6).

**Table 6:** Associations (*P* values) of known AD genes from the analysis of neuropathological phenotypes using multivariate gene-based association methods

Gene	CH	Start	Stop	Eff. SNPs^a^ (%)	VEGAS^b^				GATES					
					Univariate			Multivariate	Univariate			Multivariate		
					NP	NFT	CAA	NP+NFT +CAA O’Brien	NP	NFT	CAA	NP+NFT+CAA		
												O’Brien	TATES	MultiPhen
*CR1*	1	207,669,473	207,815,110	90.6/422 (21.5%)	0.66	0.27	0.17	0.37	0.91	0.63	0.27	0.81	0.69	0.97
*BIN1*	2	127,805,607	127,864,864	*80.3/390 (20.6%)*	3.2 × 10^−3^	1.1 × 10^−3^	0.66	7.5 × 10^−4^	7.9Ex10^−4^	3.0 × 10^−4^	0.79	1.8 × 10^−3^	5.3 × 10^−4^	0.11
*INPP5D*	2	234,054,795	234,116,549	91.6/279 (32.8%)	0.47	0.07	0.12	0.54	0.99	0.60	0.43	0.55	0.83	0.38
*CIDEC*	3	9,908,394	9,921,938	47.8/146 (32.7%)	0.45	0.57	0.58	0.34	0.76	0.06	0.59	0.57	0.14	0.70
*MEF2C*	5	88,014,058	88,199,922	88.8/338 (26.3%)	0.09	0.06	0.44	0.34	0.06	0.18	0.83	0.65	0.14	0.91
*HBEGF*	5	139,712,428	139,726,188	38.8/91 (42.6%)	0.76	0.96	0.05	0.30	0.62	0.96	0.15	0.43	0.31	0.26
*HLA-DRB5*	6	32,485,151	32,498,006	874.5/1847 (47.3%)	0.12	0.08	0.63	0.14	2.8 × 10^−3^	0.02	1.00	0.32	7.4 × 10^−3^	0.06
*CD2AP*	6	47,445,525	47,594,999	77.6/472 (16.4%)	0.24	0.46	0.31	0.27	0.48	0.67	0.64	0.66	0.71	0.63
*ZCWPW1*	7	99,998,495	100,026,302	24.5/90 (27.2%)	0.68	0.37	0.73	0.77	0.66	0.11	0.78	0.90	0.26	0.50
*PLXNA4*	7	131,808,091	132,333,447	377.7/1435 (26.3%)	0.81	0.80	0.85	0.84	0.65	0.76	0.92	0.94	0.96	0.31
*EPHA1*	7	143,088,205	143,105,985	59.1/158 (37.4%)	0.24	0.13	0.09	0.27	0.39	0.21	0.21	0.37	0.27	0.21
*PTK2B*	8	27,168,999	27,316,908	120.8/500 (24.2%)	0.10	0.63	2.9 × 10^−3^	0.07	0.31	0.80	0.01	0.13	0.03	0.05
*CLU*	8	27,454,434	27,472,328	60.8/178 (34.2%)	0.36	0.47	0.42	0.19	0.49	0.35	0.63	0.29	0.47	0.53
*PPP2CB*	8	30,643,126	30,670,352	30.3/145 (20.9%)	0.82	0.70	0.99	0.87	0.89	0.80	0.96	0.82	0.96	0.09
*USP6NL*	10	11,502,509	11,653,679	104.5/456 (22.9%)	0.40	0.12	0.85	0.86	0.55	0.54	0.97	0.53	0.79	0.13
*CELF1*	11	47,487,489	47,574,792	47.5/159 (29.9%)	0.97	0.39	0.54	0.69	0.99	0.02	0.70	0.28	0.04	0.51
*MS4A6A*	11	59,939,080	59,950,674	31.4/129 (24.4%)	0.24	0.14	0.97	0.25	0.11	0.46	0.93	0.46	0.28	0.83
*PICALM*	11	85,668,214	85,780,923	*80.0/396 (20.2%)*	0.02	2.4 × 10^−3^	0.03	8.6 × 10^−4^	0.10	5.2 × 10^−3^	0.04	2.5 × 10^−3^	0.01	0.08
*SORL1*	11	121,322,912	121,504,471	105.2/359 (29.3%)	0.20	0.08	0.92	0.51	0.22	0.13	0.96	0.08	0.34	0.18
*FERMT2*	14	53,323,989	53,417,815	72.2/260 (27.8%)	0.10	0.16	0.69	0.37	0.13	0.26	0.78	0.09	0.31	0.30
*SLC24A4*	14	92,788,925	92,967,825	210.7/766 (27.5%)	0.73	0.06	0.56	0.54	0.86	0.30	0.49	0.69	0.45	0.42
*MAPT*	17	43,971,748	44,105,700	94.2/919 (10.3%)	0.33	0.39	0.35	0.15	0.28	0.90	0.15	0.22	0.30	0.06
*TSPOAP1*	17	56,378,592	56,406,152	*65.4/193 (33.9%)*	0.50	0.68	0.29	0.40	0.76	0.52	0.18	0.14	0.36	0.05
*ABCA7*	19	1,040,102	1,065,571	116.1/339 (34.3%)		3.4 × 10^−3^	1.00	0.06	0.05	3.9 × 10^−3^	1.00	0.06	0.01	8.7 × 10^−3^
*NFIC*	19	3,359,616	3,463,603	159.0/432 (36.8%)	0.58	0.84	0.54	0.38	0.85	0.94	0.82	0.58	0.95	0.46
*APOE*	19	45,409,039	45,412,650	*57.0/145 (39.3%)*	<1.0 × 10^−6^	<1.0 × 10^−6^	<1.0 × 10^−6^	<1.0 × 10^−6^	8.2 × 10^−45^	2.9 × 10^−42^	1.7 × 10^−19^	1.6 × 10^−68^	2.1 × 10^−44^	2.5 × 10^−17^
*CASS4*	20	54,987,168	55,034,396	*64.9/215 (30.2%)*	0.07	0.02	0.10	4.4 × 10^−3^	0.25	0.08	0.33	0.04	0.19	0.01

^a^The Eff. SNPs indicates the proportion of independent SNPs out of the total number of SNPs in a gene range. The total SNPs were selected within 10 kb of both ends of the defined gene range. The genomic locations were assigned coordinates based on 1000 Genomes build 37 (hg19). Eff. SNPs attaining a *P*-value in a multivariate test that was at least one order of magnitude more significant than results for any of the univariate tests are italicized

^b^VEGAS computes *P* values using a permutation test and does not compute empirical *P* values with precision <1 × 10^−6^

**Table 7:** Novel associations (*P* values) from the analysis of neuropathological phenotypes using multivariate gene-based association methods

Gene	CH	Start	Stop	Eff. SNPs^a^ (%)	VEGAS				GATES					
					Univariate			Multivariate	Univariate			Multivariate		
					NP	NFT	CAA	NP+NFT+CAA O’Brien	NP	NFT	CAA	NP+NFT+CAA		
												O’Brien	TATES	MultiPhen
*TRAPPC12*	2	3,383,446	3,483,342	131.2/509 (25.8%)	0.09	4.0 × 10^−5^	0.5	<1.0 × 10^−6^	0.06	2.5 × 10^−5^	0.04	6.4 × 10^−5^	6.4 × 10^−5^	0.03
*TRAPPC12-AS1*	2	3,481,242	3,482,409	68.7/232 (29.6%)	2.0 × 10^−3^	3.9 × 10^−5^	5.0 × 10^−3^	<1.0 × 10^−6^	0.05	1.3 × 10^−5^	0.02	3.4 × 10^−5^	3.4 × 10^−5^	0.01
*ADI1*	2	3,501,690	3,523,350	52.8/215 (24.6%)	2.6 × 10^−3^	1.6 × 10^−5^	7.4 × 10^−4^	<1.0 × 10^−6^	0.03	1.0 × 10^−5^	0.01	2.6 × 10^−5^	2.5 × 10^−5^	6.1 × 10^−3^
*HDAC9*	7	18,126,572	19,036,993	611.5/1973 (31.0%)	0.40	0.14	0.61	0.24	0.56	0.01	5.2 × 10^−3^	6.1 × 10^−5^	0.01	3.5 × 10^−3^
*KRT2*	12	53,038,342	53,045,959	69.3/247 (28.0%)	3.7 × 10^−4^	0.11	0.01	3.3 × 10^−5^	9.5 × 10^−3^	0.21	0.17	1.5 × 10^−3^	0.02	0.03
*FLVCR2*	14	76,044,940	76,114,512	81.9/255 (32.1%)	0.02	0.01	1.3 × 10^−3^	5.8 × 10^−5^	0.15	0.11	0.02	4.2 × 10^−3^	0.04	9.4 × 10^−3^
*EXD1*	15	41,474,926	41,522,895	55.1/322 (17.1%)	9.0 × 10^−4^	6.6 × 10^−3^	0.02	7.9 × 10^−5^	0.01	0.03	0.15	4.9 × 10^−4^	0.03	0.11

^a^The Eff. SNPs indicates the proportion of independent SNPs out of the total number of SNPs in a gene range. The total SNPs were selected within 10 kb of both ends of the defined gene range. The genomic locations were assigned coordinates based on 1000 Genomes build 37 (hg19)

Three neighboring genes including *TRAPPC12*, *TRAPPC12-AS1*, and *ADI1* on chromosome 2p25.3 were identified at a gene-wide significant level (*P* < 2.7 × 10^−6^) in the MGAS for the three phenotypes (NP, NFT, and CAA) by O’Brien with VEGAS (Table 7). It should be noted that suggestively significant association with SNPs (best SNP: chr2:g:3474085C>T [rs35067331]; *P* = 5.5 × 10^−7^ in *TRAPCC12*) in the multivariate model (NP, NFT, and CAA) by the O’Brien method was observed in this region (Supplementary Fig. S1). A genome-wide sensitivity analysis showed that only *APOE* attained gene-wide significant association in all the pairings of multivariate gene-based association methods, and the *P* values of *APOE* from the multivariate association tests paired with GATES were similar for all approaches (Supplementary Table 6). In addition, we observed that the pairing of MultiPhen with GATES yielded similar association strengths (i.e., *P* values) for the new genes, except for *KRT2*, compared with the other combinations of multivariate gene-based association methods (Supplementary Table 6).

## Discussion

In this study, we proposed a multivariate gene-based association test as post-GWAS analysis by combining a multivariate association method (O’Brien, TATES, or MultiPhen) with a gene-based association method (GATES or TATES) to identify genes with pleiotropic effects on multiple phenotypes related to a complex disease. We limited the multivariate gene-based association tests of common SNPs (MAF ≥ 1%) because those methods were originally designed for common variants. We performed numerous simulations to depict the genetic (proportion of causal SNPs and independent SNPs) and phenotypic (various correlations between phenotypes in same or opposite direction) architecture of a complex disease to assess the performance of multivariate gene-based methods. Continuous phenotypes, which are normally distributed, were used for the simulation, but other types of phenotypes such as binary or survival outcomes could be analyzed with the three multivariate association methods.

Compared with other combinations of multivariate gene-based methods, selecting GATES for gene-based test and MultiPhen for multivariate test is robust for type I error and advantageous for power when the correlation between phenotypes is relatively low (*r* ≤ 0.57). However, MultiPhen requires individual-level data (genotypes and phenotypes), which is not available in most cases. This also means that MultiPhen omits samples with any missing values in any of the phenotypes, which will reduce the study power. For these cases in which MultiPhen has limited power, our simulation study suggests O’Brien with VEGAS or TATES with GATES as the second optimal multivariate gene-based method. When analyzing phenotypes with high correlations (*r* ≥ 0.86), we did not see noticeable difference in statistical power among the three multivariate association methods with GATES. O’Brien with VEGAS maintain the acceptable type I error in most scenarios for all factor loadings (or correlation between phenotypes) except for the highest factor loading (*Λ* = 0.75, *r* = 0.86). This suggests that applying VEGAS to the multivariate associations of SNPs from O’Brien method is appropriate when the phenotypic correlation is relatively low (*r* ≤ 0.57).

We also gained additional knowledge from the simulation studies with scenarios for phenotype in different directions and inclusion of phenotypes that were affected by causal variants. The O’Brien lost substantial power when the phenotypes are not correlated in same direction. Also, O’Brien yielded low power when phenotypes not affected by causal variants are included into the association tests. This may indicate that O’Brien may have better specificity compared to other multivariate methods (TATES and MultiPhen) to identify genetic loci with pleiotropy effects across the entire set of phenotypes included in a test.

In the multivariate gene-based analyses of the three neuropathological phenotypes (NP, NFT, and CAA), five known AD genes—*BIN1*, *PICALM*, *TSPOAP1*, *CASS4*, and *APOE*—reached significant association with a *P* value at least one order of magnitude smaller than each of the univariate association *P* values. The improved association in *APOE* was found in most of multivariate gene-based methods except for the method by MultiPhen with GATES. We noticed that the sample analyzed through MultiPhen was 33.2% smaller than the sample analyzed by O’Brien and TATES. This is because MultiPhen requires individual-level data (genotypes and phenotypes), while other multivariate association methods use summary statistics for each phenotype. Multiphen tests including a smaller number of subjects, for whom genotype data and information for all three phenotypes were available, yielded relatively weak associations in *APOE*. Our sensitivity analysis confirmed that the decrease significance of the *APOE* association in the pairing of MultiPhen with GATES compared to other combined methods was due to the smaller sample size. The improved association in *TSPOAP1* compared to univariate associations for the individual traits was observed only in the multivariate gene-based method by combining MultiPhen with GATES, and associations with *BIN1*, *PICALM*, and *CASS4* were detected only by O’Brien with VEGAS.

We identified gene-level significant (*P* < 2.7 × 10^−6^) associations with contiguous genes *TRAPPC12*, *TRAPPC12*-*AS1*, and *ADI1* using the multivariate gene-based approach based on O’Brien paired with VEGAS. The association findings for these three genes using other methods (O’Brien with GATES and TATES with GATES) are also moderately significant (*P* < 7.0 × 10^−5^), except for MultiPhen with GATES (*P* < 0.01). Recently, we identified associations with these same genes in a bivariate analysis of NFT and CAA using the O’Brien method with VEGAS [30]. Association of rs35067331 from the bivariate model (NFT+CAA; *P* = 5.8 × 10^−8^) was more significant than the association of rs35067331 from the trivariate model (NP+NFT+ CAA; *P* = 5.5 × 10^−7^). However, we obtained gene-wide significant evidence that these three genes have pleiotropy effects on the three neuropathological phenotypes.

In general, genes that yielded at least a moderately significant (*P* < 1.0 × 10^−4^) association using any of the multivariate gene-based methods (Table 4) contained an effective number of independent SNPs that accounted for <40% of the total number of SNPs in that region, a finding which is consistent with our observation from the simulation tests of statistical power.

It should be noted that our simulation scenarios do not represent all possible genotype–phenotype models for complex diseases. Therefore, the statistical performance (type I errors and powers) for the tested methods in this study cannot be assumed in all genome-wide multivariate gene-based studies. However, our simulation results based on diverse scenarios may indicate which multivariate association tests are most appropriate based on the phenotypic correlations.

Taken together, our comparison of multivariate gene-based association methods for detecting pleiotropy effects at the gene-level showed noticeable differences in type I error and power among the tested methods. This comparison study also provides practical and useful information for choosing a multivariate gene-based method, which can maximize power for gene-level pleiotropy analysis. For studies where individual-level data are available, MultiPhen with GATES can be the best option since this pair of methods performed best for the tested scenarios. When the proportion of missing data is high, however, we observed from the pleiotropy analysis using AD-related neuropathological traits that MultiPhen with GATES loses substantial power. This was especially true for the study of phenotypes with high correlation (*r* ≥ 0.86), when we observed no substantial difference in power among the tested methods. Therefore, we suggest O’Brien with VEGAS or TATES with GATES as alternative approaches when the proportion of missing data is high or the individual-level data are not available.

## Supplementary information

Supplemental material
