# Supplementary material for: Comparison of methods for multivariate gene-based association tests for complex diseases using common variants
Source: Eur J Hum Genet. 2019 Jan 25;27(5):811–23. doi: 10.1038/s41431-018-0327-8 (PMC6461986; doi:10.1038/s41431-018-0327-8)

**Supplementary Table 1**. Simulation design parameters.

| **Parameter**  **Names** | **Designed levels** | |
| --- | --- | --- |
|  | **Type I error rate** | **Power** |
| Factor loading (Λ) | 0.15, 0.35, 0.55, and 0.75 | |
| Independent SNPs (%) | Low (< 40%), moderate (40-60%), and high (> 60%) | |
| Causal Variants (%) | . | 5 and 15 |
| Correlation between phenotypes^*^ | . | +++^†^ and +--^‡^ |
| # of Phenotypes Affected by Causal Variants^*^ | . | 1, 2, and 3 |

^*^These parameters were applied for the simulations with the factor loading fixed at 0.55.

^†^All three phenotypes are positively correlated.

^‡^One of the three phenotypes is negatively correlated with the other two phenotypes.

**Supplementary Table 2.** Sample characteristics of three neuropathological phenotypes (neuritic plaque, neurofibrillary tangle, and cerebral amyloid angiopathy) related with Alzheimer disease

|  | **AD Cases** | **Controls** |
| --- | --- | --- |
| N | 3,135 | 463 |
| Female (%) | 1,742 (55.6) | 219 (47.3) |
| Age (SD) | 73.9 (7.9) | 78 (8.8) |
| *APOE* ε4 carriers | 1,984 | 83 |
| **Neuritic plaque** |  |  |
| None | 0 | 354 |
| Sparse | 0 | 109 |
| Moderate | 507 | 0 |
| Frequent | 2,628 | 0 |
| **Neurofibrillary tangles** |  |  |
| 0 | 0 | 70 |
| I-II | 0 | 393 |
| III-IV | 671 | 0 |
| V-VI | 2,464 | 0 |
| **Cerebral amyloid angiopathy** |  |  |
| any | 703 | 130 |
| absence | 1,531 | 39 |

**Supplementary Table 3**. Power of multivariate association methods with a gene-based association method, VEGAS.

| **Factor Loading (Λ)** | **Proportion (%) of Independent SNPs** | **Causal = 5%** | | | | | | | | | | |  | **Causal = 15%** | | | | | | | | | | |
| --- | --- | --- | --- | --- | --- | --- | --- | --- | --- | --- | --- | --- | --- | --- | --- | --- | --- | --- | --- | --- | --- | --- | --- | --- |
|  |  | **α = 0.01** | | |  | **α = 0.001** | | |  | **α = 0.0001** | | |  | **α = 0.01** | | |  | **α = 0.001** | | |  | **α = 0.0001** | | |
|  |  | **O'Brien** | **TATES** | **MultiPhen** |  | **O'Brien** | **TATES** | **MultiPhen** |  | **O'Brien** | **TATES** | **MultiPhen** |  | **O'Brien** | **TATES** | **MultiPhen** |  | **O'Brien** | **TATES** | **MultiPhen** |  | **O'Brien** | **TATES** | **MultiPhen** |
|  | 0 - 40 | 0.19 | 0.27 | 0.34 |  | 0.09 | 0.15 | 0.21 |  | 0.05 | 0.09 | 0.15 |  | 0.80 | 0.84 | 0.89 |  | 0.63 | 0.72 | 0.81 |  | 0.50 | 0.61 | 0.73 |
| 0.15 | 40 - 60 | 0.07 | 0.14 | 0.19 |  | 0.02 | 0.06 | 0.09 |  | 0.01 | 0.02 | 0.05 |  | 0.58 | 0.65 | 0.73 |  | 0.36 | 0.47 | 0.59 |  | 0.23 | 0.33 | 0.47 |
|  | 60 - 100 | 0.04 | 0.10 | 0.14 |  | 0.01 | 0.03 | 0.06 |  | 0.00 | 0.01 | 0.03 |  | 0.41 | 0.49 | 0.59 |  | 0.21 | 0.31 | 0.42 |  | 0.11 | 0.20 | 0.30 |
|  | 0 - 40 | 0.20 | 0.26 | 0.29 |  | 0.09 | 0.14 | 0.18 |  | 0.05 | 0.09 | 0.12 |  | 0.80 | 0.83 | 0.86 |  | 0.63 | 0.71 | 0.76 |  | 0.50 | 0.60 | 0.68 |
| 0.35 | 40 - 60 | 0.09 | 0.14 | 0.16 |  | 0.03 | 0.05 | 0.07 |  | 0.01 | 0.02 | 0.04 |  | 0.59 | 0.63 | 0.67 |  | 0.37 | 0.45 | 0.52 |  | 0.24 | 0.32 | 0.40 |
|  | 60 - 100 | 0.06 | 0.10 | 0.11 |  | 0.01 | 0.03 | 0.04 |  | 0.00 | 0.01 | 0.02 |  | 0.42 | 0.47 | 0.52 |  | 0.22 | 0.30 | 0.35 |  | 0.12 | 0.19 | 0.24 |
|  | 0 - 40 | 0.23 | 0.25 | 0.24 |  | 0.11 | 0.14 | 0.14 |  | 0.06 | 0.08 | 0.09 |  | 0.81 | 0.82 | 0.80 |  | 0.65 | 0.69 | 0.70 |  | 0.52 | 0.58 | 0.60 |
| 0.55 | 40 - 60 | 0.13 | 0.13 | 0.12 |  | 0.04 | 0.05 | 0.05 |  | 0.01 | 0.02 | 0.02 |  | 0.62 | 0.60 | 0.58 |  | 0.40 | 0.43 | 0.42 |  | 0.26 | 0.31 | 0.31 |
|  | 60 - 100 | 0.10 | 0.09 | 0.09 |  | 0.02 | 0.03 | 0.03 |  | 0.01 | 0.01 | 0.01 |  | 0.49 | 0.45 | 0.42 |  | 0.26 | 0.28 | 0.26 |  | 0.14 | 0.17 | 0.17 |
|  | 0 - 40 | 0.27 | 0.24 | 0.19 |  | 0.13 | 0.13 | 0.10 |  | 0.07 | 0.08 | 0.06 |  | 0.82 | 0.79 | 0.73 |  | 0.66 | 0.66 | 0.61 |  | 0.53 | 0.55 | 0.51 |
| 0.75 | 40 - 60 | 0.20 | 0.12 | 0.09 |  | 0.06 | 0.05 | 0.03 |  | 0.02 | 0.02 | 0.01 |  | 0.67 | 0.56 | 0.48 |  | 0.44 | 0.40 | 0.33 |  | 0.29 | 0.28 | 0.23 |
|  | 60 - 100 | 0.20 | 0.09 | 0.07 |  | 0.05 | 0.03 | 0.02 |  | 0.01 | 0.01 | 0.01 |  | 0.57 | 0.41 | 0.33 |  | 0.32 | 0.25 | 0.19 |  | 0.18 | 0.16 | 0.11 |

**Supplementary Table 4**. Power of multivariate association methods with a gene-based association method, GATES.

| **Factor Loading (Λ)** | **Proportion (%) of Independent SNPs** | **Causal = 5%** | | | | | | | | | | |  | **Causal = 15%** | | | | | | | | | | |
| --- | --- | --- | --- | --- | --- | --- | --- | --- | --- | --- | --- | --- | --- | --- | --- | --- | --- | --- | --- | --- | --- | --- | --- | --- |
|  |  | **α = 0.01** | | |  | **α = 0.001** | | |  | **α = 0.0001** | | |  | **α = 0.01** | | |  | **α = 0.001** | | |  | **α = 0.0001** | | |
|  |  | **O'Brien** | **TATES** | **MultiPhen** |  | **O'Brien** | **TATES** | **MultiPhen** |  | **O'Brien** | **TATES** | **MultiPhen** |  | **O'Brien** | **TATES** | **MultiPhen** |  | **O'Brien** | **TATES** | **MultiPhen** |  | **O'Brien** | **TATES** | **MultiPhen** |
|  | 0 - 40 | 0.09 | 0.19 | 0.29 |  | 0.05 | 0.10 | 0.18 |  | 0.03 | 0.06 | 0.13 |  | 0.60 | 0.76 | 0.87 |  | 0.45 | 0.61 | 0.78 |  | 0.35 | 0.49 | 0.70 |
| 0.15 | 40 - 60 | 0.02 | 0.08 | 0.15 |  | 0.01 | 0.03 | 0.07 |  | 0.00 | 0.01 | 0.04 |  | 0.29 | 0.47 | 0.64 |  | 0.17 | 0.30 | 0.49 |  | 0.11 | 0.19 | 0.38 |
|  | 60 - 100 | 0.01 | 0.04 | 0.08 |  | 0.00 | 0.01 | 0.03 |  | 0.00 | 0.00 | 0.02 |  | 0.12 | 0.27 | 0.43 |  | 0.06 | 0.13 | 0.27 |  | 0.03 | 0.07 | 0.18 |
|  | 0 - 40 | 0.09 | 0.18 | 0.24 |  | 0.05 | 0.10 | 0.15 |  | 0.03 | 0.06 | 0.10 |  | 0.61 | 0.75 | 0.82 |  | 0.46 | 0.60 | 0.72 |  | 0.36 | 0.48 | 0.63 |
| 0.35 | 40 - 60 | 0.02 | 0.07 | 0.11 |  | 0.01 | 0.03 | 0.05 |  | 0.00 | 0.01 | 0.03 |  | 0.30 | 0.46 | 0.57 |  | 0.17 | 0.29 | 0.42 |  | 0.11 | 0.19 | 0.32 |
|  | 60 - 100 | 0.01 | 0.04 | 0.06 |  | 0.00 | 0.01 | 0.02 |  | 0.00 | 0.00 | 0.01 |  | 0.12 | 0.26 | 0.35 |  | 0.06 | 0.12 | 0.21 |  | 0.03 | 0.07 | 0.14 |
|  | 0 - 40 | 0.10 | 0.18 | 0.18 |  | 0.05 | 0.09 | 0.11 |  | 0.03 | 0.05 | 0.07 |  | 0.63 | 0.74 | 0.76 |  | 0.47 | 0.59 | 0.64 |  | 0.37 | 0.48 | 0.55 |
| 0.55 | 40 - 60 | 0.03 | 0.07 | 0.08 |  | 0.01 | 0.03 | 0.03 |  | 0.00 | 0.01 | 0.02 |  | 0.32 | 0.45 | 0.47 |  | 0.19 | 0.28 | 0.32 |  | 0.12 | 0.18 | 0.23 |
|  | 60 - 100 | 0.01 | 0.04 | 0.04 |  | 0.00 | 0.01 | 0.01 |  | 0.00 | 0.00 | 0.01 |  | 0.14 | 0.25 | 0.26 |  | 0.06 | 0.12 | 0.15 |  | 0.03 | 0.06 | 0.09 |
|  | 0 - 40 | 0.12 | 0.17 | 0.13 |  | 0.06 | 0.09 | 0.07 |  | 0.03 | 0.05 | 0.05 |  | 0.65 | 0.72 | 0.67 |  | 0.49 | 0.57 | 0.54 |  | 0.38 | 0.46 | 0.44 |
| 0.75 | 40 - 60 | 0.04 | 0.07 | 0.05 |  | 0.01 | 0.03 | 0.02 |  | 0.01 | 0.01 | 0.01 |  | 0.35 | 0.43 | 0.36 |  | 0.21 | 0.27 | 0.23 |  | 0.13 | 0.17 | 0.16 |
|  | 60 - 100 | 0.02 | 0.03 | 0.02 |  | 0.00 | 0.01 | 0.01 |  | 0.00 | 0.00 | 0.00 |  | 0.16 | 0.23 | 0.18 |  | 0.07 | 0.11 | 0.09 |  | 0.04 | 0.06 | 0.06 |

**Supplementary Table 5.** Type I error rate of multivariate association methods with a gene-based association method for the phenotypes that have correlations similar to the neuropathological phenotypes.

| **Proportion of Independent SNPs (%)** | **VEGAS** | | | | | | | | | | |  | **GATES** | | | | | | | | | | |
| --- | --- | --- | --- | --- | --- | --- | --- | --- | --- | --- | --- | --- | --- | --- | --- | --- | --- | --- | --- | --- | --- | --- | --- |
|  | **α = 0.01** | | |  | **α = 0.001** | | |  | **α = 0.0001** | | |  | **α = 0.01** | | |  | **α = 0.001** | | |  | **α = 0.0001** | | |
|  | **O'Brien** | **TATES** | **MultiPhen** |  | **O'Brien** | **TATES** | **MultiPhen** |  | **O'Brien** | **TATES** | **MultiPhen** |  | **O'Brien** | **TATES** | **MultiPhen** | | **O'Brien** | **TATES** | **MultiPhen** | | **O'Brien** | **TATES** | **MultiPhen** |
| 0 - 40 | 0.01 | 0.00 | 0.01 |  | 0.001 | 0.001 | 0.001 |  | 0.0001 | 0.0007 | 0.0002 |  | 0.00 | 0.01 | 0.01 |  | 0.000 | 0.001 | 0.001 |  | 0.0000 | 0.0001 | 0.0000 |
| 40 - 60 | 0.01 | 0.00 | 0.01 |  | 0.002 | 0.000 | 0.001 |  | 0.0002 | 0.0004 | 0.0004 |  | 0.00 | 0.00 | 0.00 |  | 0.000 | 0.000 | 0.000 |  | 0.0000 | 0.0000 | 0.0000 |
| 60 - 100 | 0.01 | 0.00 | 0.02 |  | 0.002 | 0.001 | 0.002 |  | 0.0003 | 0.0005 | 0.0008 |  | 0.00 | 0.00 | 0.00 |  | 0.000 | 0.000 | 0.000 |  | 0.0000 | 0.0000 | 0.0000 |

The factor loading (Λ) and the percentage of causal variants among the total common variants in a gene were fixed at 0.55 and 15%, respectively.

**Supplementary Table 6.** Associations (P-values) for the novel genes in **Table 6** and *APOE* in models including only individuals with no missing neuropathological phenotypes using multivariate gene-based association methods.

|  |  |  |  |  | **VEGAS** | | | | |  | **GATES** | | | | | | |
| --- | --- | --- | --- | --- | --- | --- | --- | --- | --- | --- | --- | --- | --- | --- | --- | --- | --- |
|  |  |  |  |  | **Univariate** | | |  | **Multivariate** |  | **Univariate** | | |  | **Multivariate** | | |
| **Gene** | **CH** | **Start** | **Stop** | **Eff. SNPs ^†^ (%)** | **NP** | **NFT** | **CAA** |  | **NP+NFT+CAA** |  | **NP** | **NFT** | **CAA** |  | **NP + NFT + CAA** | | |
|  |  |  |  |  |  |  |  |  | **O'Brien** |  |  |  |  |  | **O'Brien** | **TATES** | **MultiPhen** |
| *TRAPPC12* | 2 | 3,383,446 | 3,483,342 | 131.2 / 509 | 0.71 | 0.14 | 0.01 |  | 0.02 |  | 0.88 | 0.19 | 0.03 |  | 0.04 | 0.07 | 0.03 |
|  |  |  |  | (25.80%) |  |  |  |  |  |  |  |  |  |  |  |  |  |
| *TRAPPC12-AS1* | 2 | 3,481,242 | 3,482,409 | 68.7 / 232 | 0.75 | 0.042 | 2.3x10^-3^ |  | 0.05 |  | 0.90 | 0.10 | 0.02 |  | 0.02 | 0.04 | 0.01 |
|  |  |  |  | (29.60%) |  |  |  |  |  |  |  |  |  |  |  |  |  |
| *ADI1* | 2 | 3,501,690 | 3,523,350 | 52.8 / 215 | 0.77 | 0.062 | 2.4x10^-4^ |  | 6.6x10^-3^ |  | 0.93 | 0.09 | 0.02 |  | 0.02 | 0.06 | 6.1x10^-3^ |
|  |  |  |  | (24.60%) |  |  |  |  |  |  |  |  |  |  |  |  |  |
| *HDAC9* | 7 | 18,126,572 | 19,036,993 | 611.5 / 1973 | 0.15 | 0.50 | 0.47 |  | 0.25 |  | 0.24 | 0.21 | 0.02 |  | 1.0x10^-3^ | 0.04 | 3.5x10^-3^ |
|  |  |  |  | (31.00%) |  |  |  |  |  |  |  |  |  |  |  |  |  |
| *KRT2* | 12 | 53,038,342 | 53,045,959 | 69.3 / 247 | 4.1x10^-3^ | 0.03 | 0.11 |  | 7.5x10^-3^ |  | 0.06 | 0.07 | 0.36 |  | 0.02 | 0.10 | 0.03 |
|  |  |  |  | (28.00%) |  |  |  |  |  |  |  |  |  |  |  |  |  |
| *FLVCR2* | 14 | 76,044,940 | 76,114,512 | 81.9 / 255 | 1.8x10^-3^ | 0.2 | 6.0x10^-3^ |  | 2.7x10^-3^ |  | 0.04 | 0.07 | 0.06 |  | 9.0x10^-3^ | 0.06 | 9.4x10^-3^ |
|  |  |  |  | (32.10%) |  |  |  |  |  |  |  |  |  |  |  |  |  |
| *EXD1* | 15 | 41,474,926 | 41,522,895 | 55.1 / 322 | 0.08 | 0.43 | 0.02 |  | 0.01 |  | 0.11 | 0.70 | 0.03 |  | 0.02 | 0.07 | 0.11 |
|  |  |  |  | (17.10%) |  |  |  |  |  |  |  |  |  |  |  |  |  |
| *APOE* | 19 | 45,409,039 | 45,412,650 | 57.0/145 | < 1.0x10^-6^ | < 1.0x10^-6^ | < 1.0x10^-6^ |  | < 1.0x10^-6^ |  | 5.2x10-9 | 2.1x10^-6^ | 4.7x10^-16^ |  | 2.5x10^-18^ | 1.1x10^-15^ | 2.5x10^-17^ |
|  |  |  |  | (39.3%) |  |  |  |  |  |  |  |  |  |  |  |  |  |

† Eff. SNPs = proportion of independent SNPs out of the total number of SNPs in tested range (within 10 kb of each end of the defined gene). Genomic coordinates were assigned based on 1000 Genomes build 37 (hg19).

**Supplementary Figure 1**. Regional plot showing association in the region including *TRAPPC12* and *ADI1* in the multivariate model of NP, NFT and CAA using O’Brien method. Each circle represents the association with a single SNP.
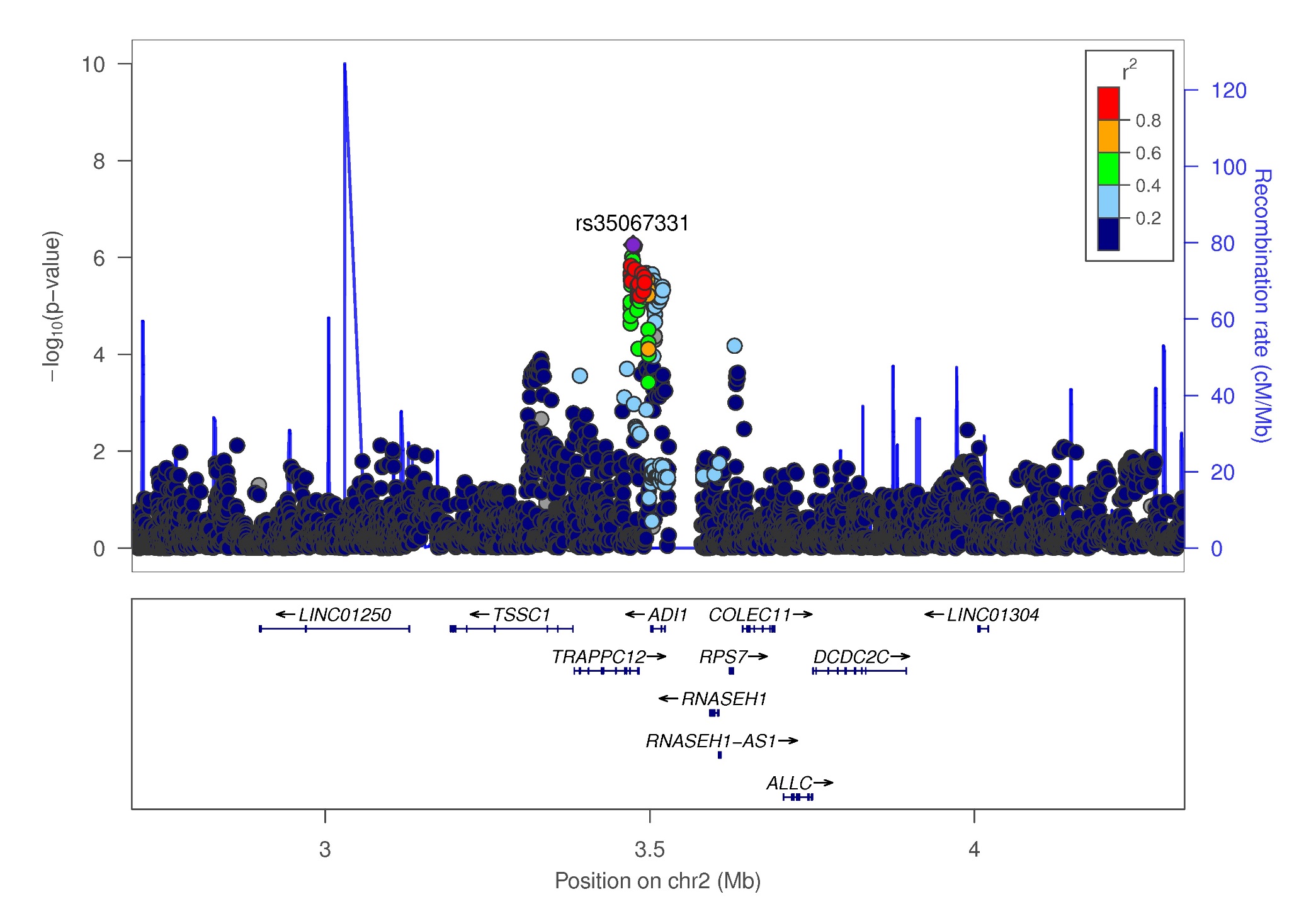

Supplement: Supplementary file 1 — Supplemental material [file 41431_2018_327_MOESM1_ESM.docx]
